# Reliability and validity of the Transthyretin Amyloidosis – Quality of Life (ATTR-QOL) Questionnaire impact scales

**DOI:** 10.1186/s41687-025-00880-7

**Published:** 2025-04-29

**Authors:** Andrew Lovley, Kristen Hsu, Kaitlin LaGasse, Isabelle Lousada, Kristen L. McCausland, Michelle K. Carty, Sabrina Rebello, Jakob B. Bjorner

**Affiliations:** 1 IQVIA Quality Metric, 225 Dyer St, 2nd Floor, Providence, RI 02903 USA; 2 Amyloidosis Research Consortium, 320 Nevada Street, Suite 210, Newton, MA 02460 USA

**Keywords:** Transthyretin (ATTR) amyloidosis, Health outcomes, Patient-reported outcome (PRO), Factor structure, Psychometric validation, Impacts, Construct validity, Item reduction, Scoring development

## Abstract

**Background:**

The diversity of disease phenotypes associated with transthyretin (ATTR) amyloidosis poses challenges for measurement of health outcomes. The Transthyretin Amyloidosis – Quality of Life (ATTR-QOL) Questionnaire is a disease-specific patient-reported outcome (PRO) measure of the symptoms and impacts of ATTR amyloidosis. The objective of this study was to evaluate the structural validity, reliability, and construct validity of the ATTR-QOL Impact scales.

**Methodology:**

This was a non-interventional, online survey study of adults with self-reported diagnosis of symptomatic ATTR amyloidosis. The survey included the ATTR-QOL and additional criterion measures. A scoring algorithm was proposed and tested. Factor structure, differential item functioning, and psychometric properties were explored.

**Results:**

The analytic sample included 233 patients. Satisfactory fit was found for a 4-factor model of disease impacts (including Daily Activities, Physical Functioning, Social/Role Functioning, and Emotional Wellbeing) and a scoring algorithm was developed. Twelve impact items were dropped from the ATTR-QOLv2 as a result of factor and differential item functioning analyses. Each scale showed evidence of satisfactory internal consistency reliability (Cronbach’s α range = 0.85–0.97) and test-retest reliability at 1 week (intraclass coefficient range = 0.84–0.97). Convergent validity was supported by correlations ≥ 0.30 between ATTR-QOL Impact scale scores and other PRO measures of related constructs. The ATTR-QOL Impact scales showed greater impact among patients with worse symptom severity, cardiac functioning, or unemployment due to ATTR amyloidosis (all p < 0.05), supporting known-groups validity.

**Conclusion:**

The ATTR-QOL is a reliable and valid measure of impacts meaningful to patients with ATTR amyloidosis. This study resulted in updates to the ATTR-QOL for item reduction and the development of a scoring algorithm. Ongoing studies are collecting data to evaluate the symptom scales of the ATTR-QOL.

**Supplementary Information:**

The online version contains supplementary material available at 10.1186/s41687-025-00880-7.

## Background

Transthyretin (ATTR) amyloidosis is a rare disease characterized by the deposition of amyloids in organ tissues throughout the body, particularly affecting the cardiac and/or nervous systems [1, 2], and causing progressive damage over time if untreated [3, 4]. Between the wild-type (ATTRwt) and over 140 known genetic variants of ATTR amyloidosis (ATTRv) [5], there is substantial diversity in the affected systems, symptoms, and patients’ overall experiences of the disease [6, 7]. The heterogeneity of disease phenotypes associated with ATTR amyloidosis poses a challenge for clinicians and researchers who aim to measure the various symptoms and impacts on health-related quality of life (HRQOL) from the patient’s perspective. The conventional use of either generic instruments or those specific to other diseases may fail to capture concepts that are important to patients with ATTR amyloidosis (or capture irrelevant concepts) and therefore undermine the validity, responsiveness, interpretability, and utility of these instruments in clinical settings [8, 9]. Survey items that attribute health impacts to other conditions, or lack a disease attribution altogether, can lack sensitivity and precision in the measurement of outcomes that patients attribute to ATTR amyloidosis.

The Transthyretin Amyloidosis – Quality of Life (ATTR-QOL) Questionnaire was recently developed as a disease-specific measure of the symptoms and impacts of ATTR amyloidosis from the patient’s perspective [10]. The decision to develop the ATTR-QOL followed calls from stakeholders for a standard measure of relevant health outcomes across types of ATTR amyloidosis, that is not only able to overcome the practical and psychometric limitations of using measures intended for the general population or for patients with other health conditions, but also fit-for-purpose in both clinical research and practice. A rigorous and iterative process was used to establish the content validity of the ATTR-QOL, involving a literature review, concept elicitation interviews with patients and clinical experts, a series of Delphi expert panel reviews, translatability assessments, informal reviews by drug developers, and cognitive debriefing interviews [10]. The objective of this study was to evaluate the reliability, structural validity, and construct validity of the ATTR-QOL Impact scales.

## Methods

### Study design and procedures

This was a non-interventional, online survey study of adults in the United States (US) with self-reported diagnosis of ATTR amyloidosis. Study participants completed an initial survey that included the ATTR-QOL and additional patient-reported outcome (PRO) measures of HRQOL, as well as questions pertaining to treatment history and clinical characteristics. A follow-up survey including only the ATTR-QOL and items evaluating change in health status was administered to study participants 1 week later.

### Study population and recruitment

Participants were recruited using convenience sampling and targeted outreach. Scripted recruitment messages were disseminated on multiple platforms managed by the Amyloidosis Research Consortium (ARC), including the organization’s website, social media, and electronic mailing lists. Individuals interested in participating were directed to an online screener used to determine eligibility and collect basic sociodemographic and clinical information. Participation in the study was limited to English-speaking adults residing in the US who reported having been diagnosed with ATTR amyloidosis by a healthcare provider, and had experienced symptoms clinically related to the condition. Informed consent was obtained from those determined to be eligible and participants were directed to the initial survey. All study materials were approved by the Western Institutional Review Board (WIRB) Copernicus Group (WCG IRB).

### Outcomes

The Transthyretin Amyloidosis Quality of Life (ATTR-QOL) Questionnaire is a comprehensive PRO that includes 32 two-part items designed to assess the frequency and severity of ATTR symptoms and 37 items designed to measure the impacts of ATTR amyloidosis on HRQOL [10]. Items refer to a “past 4 week” recall period, except for a single item about unintended weight loss/gain in the “past 12 months.” Impact scale scores ranging from 0 (No impact) to 100 (Greatest impact) are derived by averaging the item scores within each scale and transforming the score to a 0 to 100 scale. Optional items include 1 item used to capture type of ATTR amyloidosis (ATTRv or ATTRwt) and a checklist to capture self-reported past diagnosis of 11 separate comorbid conditions. The 12 optional items were administered for the initial survey only.

The SF-36v2 Health Survey (SF-36v2) is a 36-item PRO measure of general HRQOL [11]. The items measure 8 health domains: physical functioning, role limitations due to physical or emotional health, bodily pain, general health perceptions, vitality, social functioning, and mental health. Most items refer to a “past 4 week” recall period while items for the physical functioning and general health perceptions domains refer to current health status. Impacts on HRQOL are measured with 3-, 5-, or 6-point verbal rating or Likert scales. The SF-36v2 can be scored as 8 subscales on a *T*-score metric, where the US general population has a mean of 50 and a standard deviation of 10; higher scores indicate better health status. The 8 subscales can be further summarized into physical and mental component summary scores (PCS & MCS, respectively).

The Kansas City Cardiomyopathy Questionnaire – 12 Items (KCCQ-12) is a 12-item PRO measure of the impact of heart failure on HRQOL [12]. Concepts measured include physical limitations, symptom frequency, quality of life, and social limitations. Items refer to a “past 2 week” recall period except for a single item measuring satisfaction with current health status. Impacts on HRQOL are measured with 5- to 7-point verbal rating scales. The overall summary score (KCCQ-OS) and scale scores range from 0 to 100; higher scores indicate better health status and less impact of heart failure. Interpretation guidelines propose classifying KCCQ-12 scores into the following 25-point ranges: 0–24 (very poor to poor), 25–49 (poor to fair), 50–74 (fair to good), 75 or more (good to excellent) [13].

The Norfolk Quality of Life Questionnaire – Diabetic Neuropathy (Norfolk QOL-DN) is a 35-item PRO measure of the impact of neuropathy on HRQOL [14]. Concepts measured include large fiber neuropathy/physical functioning, symptoms, activities of daily living, small fiber neuropathy, and autonomic neuropathy. Most items refer to the “past 4 weeks” as the recall period while 2 items refer to current health status. Symptoms are measured as the count of affected body areas (feet, legs, hands, or arms) and impacts are measured with 5-point verbal rating or Likert scales. Scale scores are based on the sum of item responses within each domain, which are then added together to derive a total score; higher scores indicate worse neuropathic symptoms and impacts.

The 5-Level EQ-5D (EQ-5D-5L) is a 6-item PRO measure [15]. Dimensions measured included mobility, self-care, usual activities, pain/discomfort, anxiety/depression, and general health. Items refer to current health status as the recall period. Impacts are measured with 5-point verbal rating scales, while one item measures current health status with a visual analogue scale (VAS) ranging from 0 (worst health imaginable) to 100 (best health imaginable). A utility index score anchored by 0 (death) and 1 (perfect health) is derived from the responses to the 5 health dimension items.

The Patient Global Impression of Severity (PGI-S) and Change (PGI-C) are single-item PRO measures of current severity of ATTR amyloidosis and change in disease severity compared to 1 week ago, respectively. Current disease severity is measured with a 5-point verbal rating scale that ranges from 1 (absent) to 5 (very severe) and change in disease severity is measured with a 5-point Likert scale that ranges from − 2 (very much worse) to 2 (very much improved).

### Statistical analysis

Prior to analysis, the study team conducted a series of data quality checks to examine survey completion time and consistency of responses. Poor quality was indicated by: (1) SF-36v2 response consistency index (RCI) > 3; (2) completion time < 12 min; or (3) completion time < 15 min and either 3a. RCI > 2, 3b. more than one other identified inconsistent response pair, or 3c. straight-lining (same response) for all symptom items.

Sample characteristics, including socio-demographics, clinical variables, and treatment history were summarized using counts and percentages for categorical variables, and means and SDs for continuous variables. Item response means, standard deviations (SDs) and frequencies were examined.

An iterative approached was taken to evaluate the measurement model and psychometric properties of the ATTR-QOL Impact scales. The correspondence of the observed measurement model to the conceptual model proposed by the developers was assessed using confirmatory factor analysis (CFA) based on polychoric correlations and weighted least squares (WLS) estimation. Model fit was evaluated using the root mean square error of approximation (RMSEA), comparative fit index (CFI), and the Tucker-Lewis index (TLI) fit statistics. The CFA model was iteratively revised to achieve acceptable fit, defined as RMSEA ≤ 0.08, CFI ≥ 0.95, and TLI ≥ 0.95 [16]. Analysis of differential item functioning (DIF) was conducted using logistic regression to evaluate whether items performed similarly in different clinical subgroups, including ATTRwt versus ATTRv, and those with or without different types of organ involvement [17]. Evidence of DIF was defined as *p*-values < 0.05 for χ2 tests after Bonferroni-adjustment, and with differences in R2 ≥ 0.02 between a model with direct and interaction effect of subgroup on item response and a model without such effects [18]. Items showing evidence of redundancy, poor fit, and/or DIF were considered for deletion from the instrument. All subsequent analyses were based on the remaining items and scales revealed to have optimal fit.

Mokken scale analysis was conducted to determine whether item response options are monotonic, and both item- and test-scalability coefficients were assessed using a cut-off of ≥ 0.30 to indicate acceptable scalability [19]. Multi-trait analysis was performed to evaluate the hypothesized measurement model for each scale and the appropriateness of using a summated scoring algorithm. Evidence of item convergent validity was defined as item-scale correlations as ≥ 0.4 and at roughly equal magnitude within scales, and item discriminant validity was defined as higher correlations with the hypothesized scale than with other scales [20]. Evidence of satisfactory internal consistency reliability was defined as Cronbach’s α ≥ 0.70 [21]. Scale ranges were also evaluated for floor and ceiling effects, which were considered present if more than 15% achieved either highest or lowest possible score.

Psychometric evaluation of the ATTR-QOL Impact scales included descriptive analyses of scale distributions and assessment of test-retest reliability, convergent validity, and known-groups discriminant validity. The consistency of scale scores for participants with stable disease severity between the initial and 1-week follow-up surveys (defined as having the same PGI-S score at both surveys or responding “No change” on the PGI-C at 1-week follow-up survey) was evaluated for test-retest reliability using intraclass correlations (ICCs) [22]. Evidence of good test-retest reliability was defined as ICCs ≥ 0.70 [23].

Convergent and divergent validity was assessed by examining Spearman correlations between scales of the ATTR-QOL Impact domain and scales from other PRO measures. Evidence of convergent validity was defined as correlations ≥ 0.30 between ATTR-QOL scale scores and other PRO measures of related constructs. Evidence of divergent validity was based on stronger correlations between ATTR-QOL scale scores and other PRO measures of related constructs than with measures of unrelated constructs [24]. Tests for known-groups discriminant validity evaluated the ability of scales of the ATTR-QOL Impact domain to discriminate between participants belonging to subgroups known to differ in: self-reported disease severity as measured by PGI-S, employment status, disease duration, and KCCQ-OS categories. Group differences were tested using analysis of variance (ANOVA) for normally distributed scales scores, and Wilcoxon-Mann-Whitney and the Kruskal Wallis tests for non-normally distributed scale scores. Evidence of known-groups discriminant validity was defined as *p* < 0.05, indicating statistically significant differences in scale scores across subgroups [23].

## Results

### Sample characteristics

A total of 249 participants were enrolled at baseline. Of those, 234 (94%) returned to complete the 1-week follow-up survey. Data quality checks omitted 16 participants from the final analytic sample (*N* = 233), of which 219 participants (94% of the final analytic sample) completed both the baseline and the 1-week follow-up surveys. Analyses relied on the full baseline analytic sample unless otherwise noted. Socio-demographics and clinical variables for the overall study sample are reported in Table 1.

**Table 1:** Sample characteristics: Socio-demographics and clinical variables (*N* = 233)

Socio-demographics	
	Median (IQR) [Range]
**Age, years**	59 (19) [25–94]
	***N*** **(%)**
**Sex**	
Male	181 (77.7%)
Female	52 (22.3%)
**Race**	
White	208 (89.3%)
Black or African	23 (9.9%)
Asian	2 (0.9%)
American Indian or Alaska Native	1 (0.4%)
**Ethnicity**	
Hispanic or Latino	46 (19.7%)
**Education**	
At least some college	220 (94.4%)
**Employment Status** ^1^	
Not able to work due to ATTR amyloidosis	131 (56.2%)
Retired	75 (32.2%)
Currently employed	25 (10.7%)
Not employed for other reasons	2 (0.9%)
**Clinical Variables**	
	**Median (IQR) [Range]**
**Age at Diagnosis, years**	54 (20) [22–90]
**Time since Diagnosis, years**	4 (3) [0–32]
	***N*** **(%)**
**ATTR Amyloidosis Type**	
Hereditary ATTR amyloidosis (ATTRv) ^2^	132 (56.7%)
Wild-type ATTR amyloidosis (ATTRwt)	101 (43.3%)
**Genetic Variant (ATTRv sample only,** *n* ** = 132)**	
Val30Met (V30M)	45 (34.1%)
Val122Ile (V122I)	35 (26.5%)
Thr60Ala (T60A)	32 (24.2%)
Ser77TyrA (S77Y)	2 (1.5%)
Other	10 (7.6%)
Do not know	8 (6.1%)
**Affected Organs/Systems** ^3^	
Heart	182 (78.1%)
Nervous system	124 (53.2%)
Gastrointestinal system	79 (33.9%)
Kidney	44 (18.9%)
Other	15 (6.4%)
**Self-Reported Doctor Diagnoses** ^4^	
Heart failure	148 (63.5%)
Carpal tunnel syndrome	113 (48.5%)
Tendon issues	77 (33.0%)
Anxiety	66 (28.3%)
Spinal stenosis	60 (25.8%)
Depression	51 (21.9%)
Sleep apnea	40 (17.2%)
None of the above	34 (14.6%)
Malnutrition	22 (9.4%)
Stroke	18 (7.7%)
Dementia	10 (4.3%)
Seizures	3 (1.3%)
**Unintentional Loss/Gain ≥ 10 Pounds in Past Year** ^5^	
Yes	136 (58.4%)
**Ambulatory Status**	
Able to walk without assistance	150 (64.4%)
Able to walk with assistance of walker or cane	68 (29.2%)
Unable to walk	15 (6.4%)

Abbreviations: IQR, inter-quartile range; ATTR, Transthyretin Amyloidosis

^1^ Based on responses to question 11 of the ATTR-QOL

^2^ Full survey response text: *Hereditary ATTR (hATTR; also known as variant [ATTRv] or mutant [ATTRm])*. The abbreviation ATTRv is used throughout this paper based on nomenclature recommendations from the International Society of Amyloidosis

^3^ Participants could select multiple affected organs/systems

^4^ Based on responses to question 13 of the ATTR-QOL

^5^ Based on responses to question 3 of the ATTR-QOL

Current treatments for ATTR amyloidosis varied among participants (data not shown): tafamidis (*n* = 125, 53.6%), patisiran (*n* = 52, 22.3%), inotersen (*n* = 46, 19.7%), diflunisal (*n* = 38, 16.3%), tolcapone (*n* = 24, 10.3%), and others each taken by less than 10%. Few participants reported that their current treatments were provided as part of a clinical trial (*n* = 19, 8.2%), and only 5 (2.1%) participants reported no current treatment for ATTR amyloidosis. A little over 40% of participants had received a surgical treatment for ATTR amyloidosis: carpal tunnel surgery (35.2%), heart transplant (2.6%), liver transplant (1.7%), and other (11.2%).

### Patient-reported outcomes

Scores from PRO measures at the initial survey are reported in Table 2. Each PRO measure revealed evidence of disease impact across several domains of generic and condition-specific HRQOL. SF-36v2 scores for the overall study sample indicated worse generic HRQOL compared to the general population on all domains, particularly those more closely related to physical health. Among participants who reported having a diagnosis of heart failure (*n* = 148), KCCQ-12 scores generally fell in the range of “fair” (KCCQ-12 score of 50). Norfolk QOL-DN scores for the overall sample revealed notable impacts on HRQOL due to neuropathy on all domains. EQ-5D-5L scores on each dimension were on average between “slight” and “moderate” problems, and mean scores for the utility index and VAS were indicative of impaired health status.

**Table 2:** PRO measures of HRQOL at initial survey (*N* = 233)

	Mean	(SD)
SF-36v2		
Physical Functioning	36.5	(10.2)
Role Physical	39.5	(9.6)
Bodily Pain	42.6	(7.8)
General Health	39.1	(7.7)
Vitality	43.7	(8.3)
Social Functioning	38.6	(10.2)
Role Emotional	40.9	(12.1)
Mental Health	43.2	(10.4)
Physical Component Summary	38.7	(7.7)
Mental Component Summary	43.5	(10.3)
**KCCQ-12** ^1^		
Physical Limitation	51.7	(23.8)
Symptom Frequency	57.4	(18.5)
Quality of Life	47.0	(23.5)
Social Limitation	50.7	(25.2)
Overall Summary	51.7	(19.4)
**Norfolk QOL-DN**		
Activities of Daily Living	6.1	(4.9)
Autonomic Neuropathy	3.6	(2.9)
Large Fiber Neuropathy / Physical Functioning	22.5	(12.0)
Small Fiber Neuropathy	5.0	(4.1)
Symptoms	7.4	(4.8)
Total Score	44.7	(23.2)
**EQ-5D-5L**		
Mobility	2.4	(1.1)
Self-Care	2.0	(1.1)
Usual Activity	2.6	(1.0)
Pain/Discomfort	2.8	(0.9)
Anxiety/Depression	2.5	(1.1)
Utility Index	0.4	(0.3)
VAS	60.4	(14.2)

Abbreviations: EQ-5D-5L, 5-Level EQ-5D; KCCQ-12, Kansas City Cardiomyopathy Questionnaire – 12 Items; Norfolk QOL-DN, Norfolk Quality of Life– Diabetic Neuropathy; SD, standard deviation; SF-36v2, SF-36v2^®^ Health Survey; VAS, visual analog scale

^1^ Administered only to participants who reported diagnosis of heart failure (*n* = 148)

### Analysis of structural validity and item reduction of ATTR-QOL impact domain

Confirmatory factor analyses were first conducted on a prespecified 3-factor model for the ATTR-QOL Impact domain (Physical Activities, Social/Role Functioning, and Emotional Wellbeing). Modification indices suggested a 4-factor model that separated the Physical Activities factor into impacts on Daily Activities (mainly involving upper extremities) and impacts on Physical Functioning (mainly involving lower extremities). This model had satisfactory fit at the initial survey (RMSEA = 0.053, CFI = 0.985, TLI = 0.984) and at follow-up (RMSEA = 0.058, CFI = 0.981, TLI = 0.979).

Items comprising the ATTR-QOL Impact domain were evaluated for factor loadings and evidence of DIF. Twelve items were selected for deletion due to either cross-loadings or DIF. Three items also cross-loaded on the factors and/or showed evidence of DIF, but measured concepts that were deemed highly important to patients during qualitative interviews. As a result, these items were retained as single-item scales that do not contribute to the scale scores: “Feed yourself,” “Concern or guilt that you may have passed ATTR on to your child or grandchild,” and “Worry that you may not be able to access the treatments that you need for ATTR.” Subsequent analyses were based on the final 4-factor model for the ATTR-QOL Impact domain.

Mokken analysis showed acceptable scalability for all ATTR-QOL Impact domain items and scales at both initial and follow-up assessments (item and scale scalability coefficients range = 0.47–0.83). Multi-trait analysis of the items of the ATTR-QOL Impact domain provided evidence of item convergent validity with item-scale correlations ranging from 0.65 to 0.88 between items and their hypothesized scales. All but 1 item showed evidence of item discriminant validity at both initial and follow-up assessments; the item “Participate in hobbies that are important to you” correlated slightly higher with the Daily Activities scale than with the Social/Role Functioning scale (difference in *r* < 0.05).

### ATTR-QOL impact scales

Descriptive statistics for the Impact domain items are reported in Supplementary Table 1. Impact scale scores at initial survey are presented in Table 3. Mean scores ranged from 35.6 (Daily Activities) to 45.1 (Emotional Wellbeing). Scale scores were observed across the possible range of 0 to 100. Slight floor effects indicating the absence of impact in the past 4 weeks were observed for the Physical Functioning scale (17.6%) and the Daily Activities scale came close to a floor effect (14.6%); no ceiling effects were observed.

**Table 3:** ATTR-QOL impact scale scores at initial survey (*N* = 233)

	Mean (SD)	Median	Observed Range	% Floor	% Ceiling
ATTR-QOL impact scale					
Daily Activities	35.6 (28.0)	37.5	0-100	14.6	0.9
Social/Role Functioning	42.1 (27.5)	50.0	0-100	9.9	0.4
Emotional Wellbeing	45.1 (25.9)	50.0	0-100	8.2	0.4
Physical Functioning	40.6 (28.8)	50.0	0-100	17.6	1.7

Abbreviations: ATTR-QOL, Transthyretin Amyloidosis Quality of Life Questionnaire; SD, standard deviation

### Analysis of reliability of ATTR-QOL impact domain

Internal consistency and test-retest reliability estimates for the ATTR-QOL Impact scales are presented in Table 4. Tests for homogeneity of the ATTR-QOL Impact Scales showed evidence of satisfactory internal consistency reliability (α range = 0.85–0.97) and satisfactory average inter-item correlations (r range = 0.55–0.74) at initial and follow-up assessments. Each ATTR-QOL Impact scale demonstrated evidence of test-retest reliability among participants who indicated that their overall symptom severity had not changed between the initial survey and 1-week follow-up, based on responses to either the PGI-S (*N* = 126; ICC range = 0.85–0.96) or the PGI-C (*N* = 126, ICC range = 0.84–0.97).

**Table 4:** ATTR-QOL impact domain internal consistency at initial survey (*N* = 233) and Test-Retest reliability at 1-Week Follow-Up (*N* = 126)*

		Initial Survey		1-Week Follow-Up			
	# of Items	Cronbach’s α	Average Inter-Item Correlation	Cronbach’s α	Average Inter-Item Correlation	ICC (PGI-S)	ICC (PGI-C)
ATTR-QOL impact scale							
Daily Activities	8	0.94	0.68	0.94	0.66	0.93^a^	0.95
Social/Role Functioning	7	0.93	0.65	0.93	0.66	0.90	0.88
Emotional Wellbeing	3	0.85	0.65	0.86	0.66	0.85	0.84^a^
Physical Functioning	4	0.92	0.74	0.91	0.72	0.94	0.92

Abbreviations: ATTR-QOL, Transthyretin Amyloidosis Quality of Life Questionnaire; ICC, intraclass correlation coefficient; PGI-C, Patient Global Impression of Change; PGI-S, Patient Global Impression of Severity

^a^ Statistically significant (*p* < 0.05) difference in scores at initial survey and 1-week follow-up

^*^ Stable patients were defined as those who either provided the same answer at baseline and at week 1 to the PGI-S question “Overall, how would you rate the severity of your ATTR amyloidosis symptoms now?” (*N* = 126) or answered, “No change” to the PGI-C question, “Compared to 1 week ago, how would you rate the severity of your ATTR amyloidosis symptoms now?”

### Analysis of convergent validity of ATTR-QOL impact domain

Correlations between the ATTR-QOL Impact Scale scores and related PRO scales are presented in Table 5. Evidence of convergent validity was found for each ATTR-QOL Impact Scale, with correlations greater than 0.30 with measures of related constructs (bolded). Most ATTR-QOL Impact Scales demonstrated evidence of divergent validity, with higher correlations with scales that measure related constructs than with scales that are less related. The KCCQ-12 Physical Limitations scale correlated slightly higher with the ATTR-QOL Daily Activities and Social/Role Functioning scales than with the Physical Functioning scale.

**Table 5:** Correlations between ATTR-QOL impact domain scores and related PRO scores at initial survey (*N* = 233)

	ATTR-QOL Impact Scale			
PRO Scales	Daily Functioning	Social/Role Functioning	Emotional Wellbeing	Physical Functioning
Norfolk QOL-DN Small Fiber Neuropathy	**0.79**	0.62	0.54	0.53
Norfolk QOL-DN Activities of Daily Living	**0.82**	0.73	0.57	0.62
KCCQ-12 Social Limitations^a^	-0.71	**-0.70**	-0.54	-0.54
SF-36v2 – Social Function	-0.66	**-0.70**	-0.66	-0.55
SF-36v2 – Role Emotional	-0.65	**-0.68**	-0.67	-0.57
SF-36v2 – Mental Health	-0.55	-0.48	**-0.66**	-0.33
SF-36v2 – MCS	-0.61	-0.59	**-0.72**	-0.42
KCCQ-12 Physical Limitations^a^	-0.74	-0.71	-0.53	**-0.60**
SF-36v2 – Physical Function	-0.72	-0.79	-0.51	**-0.77**
SF-36v2 – PCS	-0.49	-0.66	-0.33	**-0.64**

*Abbreviations* ATTR-QOL, Transthyretin Amyloidosis Quality of Life Questionnaire; KCCQ-12, Kansas City Cardiomyopathy Questionnaire – 12 item; MCS, mental component summary; Norfolk QOL-DN, Norfolk Quality of Life – Diabetic Neuropathy; PCS, physical component summary; PGI-S, Patient Global Impression of Severity; PRO, patient-reported outcome; SF-36v2, SF-36v2^®^ Health Survey

Note Bolded cells represent variables that were hypothesized to be more highly correlated as evidence for convergent validity. Due to the directionality of the scales, correlations between ATTR-QOL Impact Scale scores with Norfolk QOL-DN scales are expected to be positive and correlations between all other scales are expected to be negative

^a^ The KCCQ-12 was only administered to patients who indicated they had a diagnosis of heart failure (*N* = 148)

### Analysis of Known-Groups discriminant validity of ATTR-QOL impact domain

Median scores for ATTR-QOL Impact scales, stratified by levels of symptom severity (PGI-S), time since diagnosis (duration of disease), cardiac functioning (KCCQ-OS) among participants with heart failure, and employment status are presented in Table 6. Evidence of known-groups discriminant validity was found for all ATTR-QOL Impact scales, with statistically significant differences across subgroups and higher (greater impact) scores among participants with worse symptom severity, cardiac functioning, or unemployment due to ATTR amyloidosis. ATTR-QOL Impact scale scores did not discriminate between participants based on duration of disease.

**Table 6:** ATTR-QOL impact scale Known-Groups discriminant validity (*N* = 233)

	ATTR-QOL Impact Scales - Median (IQR)			
Stratification	Daily Activities	Social/Role Functioning	Emotional Wellbeing	Physical Functioning
PGI-S Symptom Severity				
Absent/Mild (*n* = 95)	9.4 (46.9)	14.3 (53.6)	33.3 (33.3)	6.3 (43.8)
Moderate (*n* = 97)	43.8 (46.9)	57.1 (35.7)	50.0 (41.7)	50.0 (25.0)
Severe/Very Severe (*n* = 41)	62.5 (18.8)	64.3 (17.9)	58.3 (16.7)	62.5 (18.8)
*p-value*	< 0.001*	< 0.001*	*< 0.001*	< 0.001*
Time Since Diagnosis^a^				
0–3 years (*n* = 78)	18.8 (56.3)	50.0 (50.0)	50.0 (41.7)	43.8 (56.3)
4 years (*n* = 48)	46.9 (53.1)	57.1 (46.4)	50.0 (37.5)	43.8 (46.9)
5–6 years (*n* = 69)	43.8 (50.0)	50.0 (50.0)	50.0 (41.7)	50.0 (56.3)
7–32 years (*n* = 38)	43.8 (59.4)	55.4 (42.9)	50.0 (50.0)	56.3 (50.0)
*p-value*	*0.124*	*0.748*	*0.828*	*0.716*
Cardiac Functioning^b^				
Poor/Fair (*n* = 86)	59.4 (15.6)	64.3 (14.3)	66.7 (25.0)	62.5 (18.8)
Good (*n* = 40)	7.8 (42.2)	37.5 (35.7)	37.5 (41.7)	43.8 (50.0)
Excellent (*n* = 22)	0.0 (6.3)	1.8 (21.4)	16.7 (33.3)	6.3 (18.8)
*p-value*	< 0.001*	< 0.001*	< 0.001*	< 0.001*
Employment Status				
Employed (*n* = 25)	6.3 (18.8)	17.9 (50.0)	25.0 (41.7)	25.0 (62.5)
Not employed due to ATTR amyloidosis (*n* = 131)	56.3 (28.1)	60.7 (21.4)	58.3 (25.0)	56.3 (25.0)
*p-value*	*< 0.001*	*< 0.001*	*< 0.001*	*0.036*

Abbreviations: ATTR-QOL, Transthyretin Amyloidosis Quality of Life Questionnaire; IQR, interquartile range; PGI-S, Patient Global Impression of Severity

^*^ Pairwise comparisons between neighboring groups were also significant (*p* < 0.05); tested only for stratifications with 3 or more groups where overall test was significant (*p* < 0.05)

^a^ Time since diagnosis is stratified by quartiles

^b^ Cardiac functioning was based on categorization of the KCCQ-OS using interpretation thresholds recommended by the developers; The KCCQ-12 was only administered to patients who indicated they had a diagnosis of heart failure (*N* = 148)

## Discussion

This was the first study to evaluate the psychometric properties of the ATTR-QOL as a PRO measure of the impacts of ATTR amyloidosis on HRQOL. Factor analysis revealed a 4-factor model to have superior fit to the ATTR-QOL Impact domain data than the originally proposed 3-factor conceptual model. Twelve impact items were dropped from the ATTR-QOLv2 as a result of factor and DIF analyses, and three items (‘feed yourself’, ‘concern for passing ATTR on to child’, and ‘worry may not be able to access treatment’) were retained as single-item Impact scales, as these items were judged to be important despite relatively lower factor loadings or some evidence of DIF. The 4 separate scales of the ATTR-QOL Impact domain showed evidence of scalability and unidimensionality, minimal floor and ceiling effects, and convergent and discriminant validity at both the item- and scale-level.

Although other efforts are underway to create and validate disease-specific PROs for patients with ATTR amyloidosis, the ATTR-QOL appears to provide the broadest conceptual coverage of symptoms and impacts meaningful to patients with ATTR amyloidosis and have the broadest range of applicability [25, 26]. The decision to create a single instrument that captures symptoms and impacts across both types of amyloidosis and avoids categorizing patients a priori (by type or subtype) was based on input from patients and experts and is supported by research indicating the presence of diverse symptomology and phenotypes even within patients with ATTRwt [10, 27, 28]. Developing a PRO that is fit-for-purpose across a broad range of contexts can improve the field’s understanding of the condition by capturing the true variability, experiences, and enduring unmet need associated with ATTR amyloidosis using a standardized, but efficient approach. Strong correlations between the ATTR-QOL Impact scale scores and related domains of the SF-36v2, KCCQ-12, and Norfolk QOL-DN confirm the ability of the ATTR-QOL to capture the impacts measured by these instruments, with the additional precision provided by item content that is tailored to the experiences of patients with ATTR amyloidosis and attributed to their disease.

Psychometric validation of the ATTR-QOL is an ongoing process, alternating between the evaluation and data-driven adaptation of the instrument. Each evaluation adds information on the reliability and validity of the measure, particularly in specific contexts. Although this study represents an important first step in gathering psychometric evidence of the ATTR-QOL, the analyses were somewhat limited in scope (i.e., centered on structural validity, reliability, and convergent/discriminant validity). Psychometric properties such as ability to detect change were not evaluated and clinical biomarkers were not part of the data. Secondly, ATTR amyloidosis is a rare disease with a wide range of phenotypes, and generalizability across patients with ATTR amyloidosis is limited by the final composition of the sample. For example, 64% of participants in this study reported being able to walk unassisted (Cotinhou’s stage 1 of ambulatory disability) and 84% reported being diagnosed within the past 6 years, suggesting that many were relatively early in the course of their disease and possibly experiencing less symptom burden or impacts on HRQOL relative to those whose disease has progressed to later stages. The sample composition is likely the reason for the moderate floor effects seen for items on ability to walk independently, bathe, dress, or feed themselves. However, these items are important for patients with more advanced disease and no major floor effects were seen on scale level. Furthermore, online administration of the survey may have limited participation to those who have the means and literacy to access the internet and complete the surveys, as well as to those who feel healthy enough to participate. Participants recruited through their affiliation with a patient advocacy group may also have a more active role in understanding and managing their disease, which may further restrict the generalizability of the results.

This study and the original development and content validation study [10] together provide evidence that the ATTR-QOL Impact scales are valid across types of ATTR amyloidosis, including ATTRwt and multiple variants of ATTRv. Ongoing studies will evaluate the sensitivity of the next version of the survey (ATTR-QOLv2) Impact scales, in particular their sensitivity to disease progression and in response to treatment. Also, additional data is being collected to evaluate the possibility for Symptom scales and to test the performance of the instrument among patients with other genetic variants and levels of disease severity.

## Conclusion

The ATTR-QOL is a PRO measure of the symptoms and impacts of ATTR amyloidosis on HRQOL that is designed to be appropriate for all types of ATTR amyloidosis, and useful for clinicians and researchers alike. Analyses of the Impact domain provide a 4-factor measurement model and lead to recommendations for item-reduction for the next version of the survey (ATTR-QOLv2). Finally, results showed evidence of reliability and validity for the ATTR-QOL Impact scales. Further research is needed to explore the validity of symptom scales, evaluate the sensitivity of ATTR-QOL in longitudinal studies, and test the performance of the instrument in other clinical subgroups of ATTR amyloidosis.

## Electronic supplementary material

Below is the link to the electronic supplementary material.

Supplementary Material 1

## Data Availability

Raw data are not publicly available due to confidentiality and consent limitations. Additional study information is available from the corresponding author upon reasonable request.

## References

[1] Schwartzlow C, Kazamel M (2020) Hereditary transthyretin amyloidosis: clinical presentation and management updates. J Clin Neuromuscul Dis 21(3):144–156. 10.1097/CND.0000000000000270 32073460 10.1097/CND.0000000000000270

[2] Griffin JM, Rosenblum H, Maurer MS (2021) Pathophysiology and therapeutic approaches to cardiac amyloidosis. Circul Res 128(10):1554–1575. 10.1161/CIRCRESAHA.121.318187 10.1161/CIRCRESAHA.121.318187 PMC8561842 33983835

[3] Ponti L, Hsu K, Damy T et al (2023) Burden of untreated transthyretin amyloid cardiomyopathy on patients and their caregivers by disease severity: results from a multicenter, non-interventional, real-world study. Front Cardiovasc Med 10:1238843. 10.3389/fcvm.2023.1238843 37711563 10.3389/fcvm.2023.1238843 PMC10497948

[4] Yarlas A, Gertz MA, Dasgupta NR et al (2019) Burden of hereditary transthyretin amyloidosis on quality of life. Muscle Nerve 60(2):169–175. 10.1002/mus.26515 31093980 10.1002/mus.26515 PMC6771567

[5] Rowczenio DM, Noor I, Gillmore JD et al (2014) Online registry for mutations in hereditary amyloidosis including nomenclature recommendations. Hum Mutat 35(9):E2403–E2412. 10.1002/humu.22619 25044787 10.1002/humu.22619

[6] Lovley A, Raymond K, Guthrie SD, Pollock M, Sanchorawala V, White MK (2021) Patient-reported burden of hereditary transthyretin amyloidosis on functioning and well-being. J Patient-Reported Outcomes 5(1):3. 10.1186/s41687-020-00273-y 10.1186/s41687-020-00273-y PMC7790957 33411323

[7] Rintell D, Heath D, Braga Mendendez F et al (2021) Patient and family experience with transthyretin amyloid cardiomyopathy (ATTR-CM) and polyneuropathy (ATTR-PN) amyloidosis: results of two focus groups. Orphanet J Rare Dis 16(1):70. 10.1186/s13023-021-01706-7 33557882 10.1186/s13023-021-01706-7 PMC7869246

[8] Ware JE, Gandek B, Allison J (2016) The validity of disease-specific quality of life attributions among adults with multiple chronic conditions. Int J Stat Med Res 5(1):17–40. 10.6000/1929-6029.2016.05.01.3 27087882 10.6000/1929-6029.2016.05.01.3 PMC4831653

[9] Terwee CB, Prinsen CAC, Chiarotto A et al (2018) COSMIN methodology for evaluating the content validity of patient-reported outcome measures: A Delphi study. Qual Life Res 27(5):1159–1170. 10.1007/s11136-018-1829-0 29550964 10.1007/s11136-018-1829-0 PMC5891557

[10] O’Connor M, Hsu K, Broderick L et al (2023) The transthyretin amyloidosis– quality of life (ATTR-QOL) questionnaire: development of a conceptual model and disease-specific patient-reported outcome measure. Patient Relat Outcome Measures 14:213–222. 10.2147/PROM.S411721 10.2147/PROM.S411721 PMC10335277 37441025

[11] Maruish ME (2011) User’s manual for the SF-36v2 health survey, 3rd edn. QualityMetric, Inc.

[12] Spertus JA, Jones PG (2015) Development and validation of a short version of the Kansas City cardiomyopathy questionnaire. Circulation: Cardiovasc Qual Outcomes 8(5):469–476. 10.1161/CIRCOUTCOMES.115.001958 10.1161/CIRCOUTCOMES.115.001958 PMC4885562 26307129

[13] Spertus JA, Jones PG, Sandhu AT, Arnold SV (2020) Interpreting the Kansas City cardiomyopathy questionnaire in clinical trials and clinical care: JACC state-of-the-art review. J Am Coll Cardiol 76(20):2379–2390. 10.1016/j.jacc.2020.09.542 33183512 10.1016/j.jacc.2020.09.542

[14] Vinik EJ, Hayes RP, Oglesby A et al (2005) The development and validation of the Norfolk QOL-DN, a new measure of patients’ perception of the effects of diabetes and diabetic neuropathy. Diabetes Technol Ther 7(3):497–508. 10.1089/dia.2005.7.497 15929681 10.1089/dia.2005.7.497

[15] Janssen MF, Pickard AS, Golicki D et al (2013) Measurement properties of the EQ-5D-5L compared to the EQ-5D-3L across eight patient groups: A multi-country study. Qual Life Res 22(7):1717–1727. 10.1007/s11136-012-0322-4 23184421 10.1007/s11136-012-0322-4 PMC3764313

[16] Hu L, Bentler PM (1999) Cutoff criteria for fit indexes in covariance structure analysis: conventional criteria versus new alternatives. Struct Equation Modeling: Multidisciplinary J 6(1):1–55. 10.1080/10705519909540118

[17] Zumbo B (1999) A handbook on the theory and methods of differential item functioning (DIF): logistic regression modeling as a unitary framework for binary and Likert-type (ordinal) item scores. National Defense Headquarters

[18] Bjorner JB, Kosinski M, Ware JE Jr (2003) Calibration of an item pool for assessing the burden of headaches: an application of item response theory to the headache impact test (HIT™). Qual Life Res 12:913–933. 10.1023/a:1026163113446 14651412 10.1023/a:1026163113446

[19] van der Ark LA (2012) New developments in Mokken scale analysis in R. J Stat Softw 48(5). 10.18637/jss.v048.i05

[20] Ware JE, Harris WJ, Gandek B, Rogers BW, Reese PR (1996) MAP-R for Windows: Multitrait/Multi-Item analysis Program - Revised user’s guide. Health Assessment Lab, Incorporated

[21] Bland JM, Altman DG (1997) Cronbach’s alpha. BMJ 314(7080):572. 10.1136/bmj.314.7080.572 9055718 10.1136/bmj.314.7080.572 PMC2126061

[22] McGraw KO, Wong SP (1996) Forming inferences about some intraclass correlation coefficients. Psychol Methods 1(1):30–46. 10.1037/1082-989X.1.1.30

[23] de Vet HCW, Terwee CB, Mokkink LB, Knol DL (2011) Measurement in medicine: a practical guide. Cambridge University Press

[24] Campbell DT, Fiske DW (1959) Convergent and discriminant validation by the multitrait-multimethod matrix. Psychol Bull 56(2):81–105. 10.1037/h0046016 13634291

[25] Kharoubi M, Bézard M, Broussier A et al (2023) Amylo-AFFECT-QOL, a self-reported questionnaire to assess health-related quality of life and to determine the prognosis in cardiac amyloidosis. Front Cardiovasc Med 10:1124660. 10.3389/fcvm.2023.1124660 36998975 10.3389/fcvm.2023.1124660 PMC10043221

[26] Aimo A, Teresi L, Castiglione V et al (2023) Patient-reported outcome measures for transthyretin cardiac amyloidosis: the ITALY study. Amyloid 1–10. 10.1080/13506129.2023.2254451 10.1080/13506129.2023.2254451 37668548

[27] Wajnsztajn Yungher F, Kim A, Boehme A et al (2020) Peripheral neuropathy symptoms in wild type transthyretin amyloidosis. J Peripheral Nerv Syst 25(3):265 10.1111/jns.12403 32627282

[28] Sabbour H, Hasan KY, Al Badarin F et al (2021) From clinical clues to final diagnosis: the return of detective work to clinical medicine in cardiac amyloidosis. Front Cardiovasc Med 8:644508. 10.3389/fcvm.2021.644508 34262948 10.3389/fcvm.2021.644508 PMC8274453

