# Supplementary material for: Reliability and validity of the Transthyretin Amyloidosis – Quality of Life (ATTR-QOL) Questionnaire impact scales
Source: J Patient Rep Outcomes. 2025 Apr 29;9:44. doi: 10.1186/s41687-025-00880-7 (PMC12040782; doi:10.1186/s41687-025-00880-7)
Supplement: Supplementary file 1 — Supplementary Material 1 [file 41687_2025_880_MOESM1_ESM.docx]

**Supplementary Table 1.** ATTR-QOL Impact Domain Item Responses (N=233)

|  |  |  |  | **Not at all** | | **A little** | | **Some** | | **A lot** | | **Completely; I cannot do it at all / Extremely** | |
| --- | --- | --- | --- | --- | --- | --- | --- | --- | --- | --- | --- | --- | --- |
| **Item Text** | **N** | **Mean** | **SD** | **N** | **%** | **N** | **%** | **N** | **%** | **N** | **%** | **N** | **%** |
| Problems with balance impacted ability to do regular daily activities | 233 | 1.7 | 1.1 | 47 | 20.2% | 45 | 19.3% | 74 | 31.8% | 59 | 25.3% | 8 | 3.4% |
| Stand up independently from a chair | 233 | 1.4 | 1.2 | 71 | 30.5% | 64 | 27.5% | 50 | 21.5% | 41 | 17.6% | 7 | 3.0% |
| Stand for 5 minutes independently (without support) | 233 | 1.3 | 1.2 | 85 | 36.5% | 40 | 17.2% | 63 | 27.0% | 35 | 15.0% | 10 | 4.3% |
| Walk independently (without support) | 233 | 1.4 | 1.3 | 95 | 40.8% | 26 | 11.2% | 43 | 18.5% | 61 | 26.2% | 8 | 3.4% |
| Walk up or down hills | 233 | 2.2 | 1.4 | 45 | 19.3% | 31 | 13.3% | 37 | 15.9% | 68 | 29.2% | 52 | 22.3% |
| Walk on uneven ground | 233 | 1.5 | 1.1 | 56 | 24.0% | 62 | 26.6% | 65 | 27.9% | 41 | 17.6% | 9 | 3.9% |
| Climb up a few steps (3-5 steps) | 233 | 1.3 | 1.2 | 78 | 33.5% | 53 | 22.7% | 61 | 26.2% | 30 | 12.9% | 11 | 4.7% |
| Climb up a flight of stairs (10 or more steps) | 233 | 1.9 | 1.3 | 53 | 22.7% | 43 | 18.5% | 46 | 19.7% | 65 | 27.9% | 26 | 11.2% |
| Walk 100 yards | 233 | 1.5 | 1.3 | 75 | 32.2% | 38 | 16.3% | 67 | 28.8% | 38 | 16.3% | 15 | 6.4% |
| Walk half a mile | 233 | 1.9 | 1.5 | 68 | 29.2% | 26 | 11.2% | 48 | 20.6% | 50 | 21.5% | 41 | 17.6% |
| Walk for 5 minutes | 233 | 1.5 | 1.3 | 76 | 32.6% | 34 | 14.6% | 67 | 28.8% | 45 | 19.3% | 11 | 4.7% |
| Walk for 20 minutes | 233 | 2.2 | 1.4 | 46 | 19.7% | 31 | 13.3% | 28 | 12.0% | 87 | 37.3% | 41 | 17.6% |
| Participate in physical activities | 233 | 2.1 | 1.3 | 33 | 14.2% | 42 | 18.0% | 53 | 22.7% | 75 | 32.2% | 30 | 12.9% |
| Do household chores | 233 | 1.5 | 1.2 | 48 | 20.6% | 73 | 31.3% | 62 | 26.6% | 36 | 15.5% | 14 | 6.0% |
| Bathe yourself | 233 | 1.3 | 1.3 | 94 | 40.3% | 46 | 19.7% | 39 | 16.7% | 42 | 18.0% | 12 | 5.2% |
| Pull a shirt down over your head or pull up pants | 233 | 1.3 | 1.3 | 90 | 38.6% | 53 | 22.7% | 42 | 18.0% | 36 | 15.5% | 12 | 5.2% |
| Button a shirt or pull up zippers | 233 | 1.3 | 1.3 | 96 | 41.2% | 41 | 17.6% | 48 | 20.6% | 32 | 13.7% | 16 | 6.9% |
| Feed yourself | 233 | 1.1 | 1.3 | 107 | 45.9% | 47 | 20.2% | 37 | 15.9% | 31 | 13.3% | 11 | 4.7% |
| Complete tasks with your hands | 233 | 1.4 | 1.2 | 66 | 28.3% | 68 | 29.2% | 45 | 19.3% | 41 | 17.6% | 13 | 5.6% |
| Cook or prepare everyday meals | 233 | 1.4 | 1.4 | 84 | 36.1% | 47 | 20.2% | 41 | 17.6% | 41 | 17.6% | 20 | 8.6% |
| Run household errands | 233 | 1.5 | 1.3 | 70 | 30.0% | 56 | 24.0% | 43 | 18.5% | 41 | 17.6% | 23 | 9.9% |
| Drive or use public transportation | 233 | 1.5 | 1.5 | 88 | 37.8% | 39 | 16.7% | 43 | 18.5% | 29 | 12.4% | 34 | 14.6% |
| Participate in hobbies that are important to you | 233 | 1.6 | 1.2 | 62 | 26.6% | 41 | 17.6% | 61 | 26.2% | 58 | 24.9% | 11 | 4.7% |
| Participate in fun or recreational activities with other people | 233 | 1.7 | 1.2 | 57 | 24.5% | 41 | 17.6% | 65 | 27.9% | 57 | 24.5% | 13 | 5.6% |
| Visit with family and friends | 233 | 1.4 | 1.3 | 81 | 34.8% | 47 | 20.2% | 42 | 18.0% | 56 | 24.0% | 7 | 3.0% |
| Get together with friends somewhere other than your home | 233 | 1.5 | 1.3 | 74 | 31.8% | 50 | 21.5% | 43 | 18.5% | 45 | 19.3% | 21 | 9.0% |
| Attend gatherings with family somewhere other than your home | 233 | 1.4 | 1.3 | 73 | 31.3% | 59 | 25.3% | 42 | 18.0% | 45 | 19.3% | 14 | 6.0% |
| Participate in your family the way you would like | 233 | 1.7 | 1.2 | 47 | 20.2% | 66 | 28.3% | 54 | 23.2% | 47 | 20.2% | 19 | 8.2% |
| Take care of your family | 233 | 1.6 | 1.4 | 74 | 31.8% | 41 | 17.6% | 53 | 22.7% | 35 | 15.0% | 30 | 12.9% |
| Plan outings, events, or travel within the next month | 233 | 1.8 | 1.3 | 60 | 25.8% | 37 | 15.9% | 55 | 23.6% | 56 | 24.0% | 25 | 10.7% |
| Plan outings, events, or travel for the future | 233 | 1.9 | 1.4 | 62 | 26.6% | 25 | 10.7% | 49 | 21.0% | 73 | 31.3% | 24 | 10.3% |
| ATTR interfered with your relationships with loved ones | 233 | 1.7 | 1.2 | 46 | 19.7% | 51 | 21.9% | 72 | 30.9% | 52 | 22.3% | 12 | 5.2% |
| Fear or anxiety related to ATTR | 233 | 1.8 | 1.2 | 38 | 16.3% | 64 | 27.5% | 58 | 24.9% | 54 | 23.2% | 19 | 8.2% |
| Worry about the future | 233 | 1.9 | 1.1 | 23 | 9.9% | 72 | 30.9% | 68 | 29.2% | 53 | 22.7% | 17 | 7.3% |
| Sadness or depression about having ATTR | 233 | 1.8 | 1.2 | 45 | 19.3% | 58 | 24.9% | 58 | 24.9% | 52 | 22.3% | 20 | 8.6% |
| Concern or guilt that you may have passed ATTR on to your child or grandchild | 233 | 1.6 | 1.4 | 81 | 34.8% | 38 | 16.3% | 40 | 17.2% | 49 | 21.0% | 25 | 10.7% |
| Worry that you may not be able to access the treatments that you need for ATTR | 233 | 1.8 | 1.2 | 45 | 19.3% | 49 | 21.0% | 63 | 27.0% | 56 | 24.0% | 20 | 8.6% |
| Work your regularly scheduled number of hours^1^ | 25 | 0.9 | 1.0 | 10 | 40.0% | 11 | 44.0% | 2 | 8.0% | 1 | 4.0% | 1 | 4.0% |
| Be productive at work^1^ | 25 | 1.1 | 1.0 | 9 | 36.0% | 6 | 24.0% | 8 | 32.0% | 2 | 8.0% | 0 | 0.0% |

Abbreviations: ATTR, transthyretin amyloidosis; ATTR-QOL, Transthyretin Amyloidosis Quality of Life Questionnaire; SD, standard deviation

^1^ Administered only to participants who reported current employment (n=25)
